# Effects of *Artemisia asiatica ex* on *Akkermansia muciniphila* dominance for modulation of Alzheimer’s disease in mice

**DOI:** 10.1371/journal.pone.0312670

**Published:** 2024-10-28

**Authors:** Mijung Lee, Kwang-Sung Ahn, Manho Kim

**Affiliations:** 1 Department of Neurology, Biomedical Research Institute, Seoul National University Hospital, Seoul, South Korea; 2 Functional Genome Institute, PDXen. Biosystem Co., Gyeongi-do, South Korea; 3 Neuroscience Dementia Research Institute, Seoul National University College of Medicine, Seoul, South Korea; 4 Protein Metabolism Medical Research Center, College of Medicine, Seoul National University Hospital, Seoul, South Korea; Wuhan Polytechnic University, CHINA

## Abstract

The gut microbiome influences neurological disorders through bidirectional communication between the gut and the brain, i.e., the gut-brain axis. *Artemisia asiatica ex*, an extract of *Artemisia asiatica* Nakai (Stillen®, DA-9601) has been reported to improve depression by increasing brain-derived neurotropic factor. Therefore, we hypothesized that DA-9601 can be a potential therapeutic candidate for Alzheimer’s disease (AD) acting through the gut-brain axis. Four groups of Tg2576 mice were used as the animal model for AD: wild type mice (n = 6), AD mice (n = 6), and DA-9601-administered AD mice given dosages of 30mg/kg/day (DA_30mg; n = 6) or 100mg/kg/day (DA_100mg; n = 6). Microglial activation, blood‒brain barrier integrity, amyloid beta accumulation, cognitive behavior, and changes in the gut microbiome were analyzed. DA-9601 improved the cognitive behavior of mice (DA_30mg **p<0.01; DA_100mg **p<0.01) and reduced amyloid beta accumulation (DA_30mg ***p<0.001; DA_100mg **p<0.01). Increased Iba-1 and upregulation of claudin-5 (DA_30mg *p<0.05) and occludin (DA_30mg **p<0.01; DA_100mg ***p<0.001) indicated altered microglial activation and improved blood‒brain barrier integrity. *Akkermansia muciniphila* was dramatically increased by DA-9601 administration (DA_30mg 47%; DA_100mg 61%). DA-9601 improved AD pathology with *Akkermansia muciniphila* dominance in the gut microbiome in a mouse model of AD, inferring that DA-9601 can affect AD through the gut-brain axis.

## Introduction

The main pathology of Alzheimer’s disease (AD) is the accumulation of amyloid beta (Aβ) in the central nervous system. Mitochondrial dysfunction, oxidative stress, late apoptotic cell death, and microglial activation have been proposed to cause aging of the immune system [1–4]. Progressive brain damage with cognitive decline is the most serious challenge in the treatment of AD [5, 6]. Previous studies have focused on developing therapeutic agents targeting the central nervous system [7, 8]. However, an altered gut microbiome may be associated with neurological disorders such as AD, Parkinson’s disease, seizures, and multiple sclerosis [9–12].

*Artemisia asiatica ex* is an extract of *Artemisia asiatica* Nakai, with Eupatilin as its main active component (Stillen®, DA-9601). This extract is used to treat gastric mucosal ulcers and inflammation [13, 14]. It has been shown to restore gastric mucosal damage caused by nonsteroidal anti-inflammatory drugs (NSAIDs) or *Helicobacter pylori*. It inhibits the cytochrome 2E1 ethanol metabolic enzyme stimulated by alcohol and protects the intestinal lining by blocking the action of pro-inflammatory mediators [13, 15]. DA-9601 has also been reported to alleviate depression by increasing brain-derived neurotropic factor in the hippocampus via regulation of proinflammatory mediators [16]. Therefore, DA-9601 may represent a potential therapeutic candidate for AD, potentially influencing the gut-brain axis.

The gut microbiome comprises more than 95% of the total bacteria in the body. These microbes influence intestinal mucosal lymphoid tissue, thereby altering the immune response through cytokine signaling [17–19]. The immune response in the brain is affected by bidirectional communication via signals secreted from the gut microbiome [20, 21]. The gastrointestinal (GI) tract microbiome is primarily composed of Bacteroidetes (~48%), Firmicutes (~51%), and smaller proportions of Proteobacteria, Verrucomicrobia, Fusobacteria, Cyanobacteria, Actinobacteria, and Spirochetes (~1%) [22]. Various metabolites produced by the gut microbiome are known to modulate both the central and peripheral nervous systems, either directly or indirectly, by altering in (blood‒brain barrier) BBB permeability [22–25].

*Akkermansia muciniphila* is associated with human health and can be identified through metagenomic analysis of the human intestine. It is the only representative of Verrucomicrobia in the human gut microbiome that can be cultured [26]. *Akkermansia muciniphila* levels are decreased in the AD mouse model [27]. This bacterium has the capacity to repair the damaged epithelial barrier, thereby providing protection against endotoxemia [26, 28, 29]. Administration of lactic acid bacteria and fecal transplantation have been attempted as therapeutic strategies to improve the gut microbiome environment [30–32]. These interventions have been shown to increase the production of short-chain fatty acids (SCFAs), decrease microglial activation induced by harmful bacterial products such as lipopolysaccharides (LPS) and amyloids, and reduce chronic inflammatory responses in the brain by restoring BBB function in AD pathology [33–35].

Here, we investigated whether DA-9601 could ameliorate AD through alterations in the gut microbiome. Cognitive function, Aβ accumulation, BBB integrity, and microglial activation were monitored as well as changes in gut microbiome diversity in response to administration of DA-9601. The reported characteristics of DA-9601 suggest that its protective effects against inflammation activate shortly after administration. [36–40] Therefore, we aimed to assess the effects of DA-9601 from the early stages of the AD by administering DA-9601 to Tg2576 mice aged 9 months over a period of 2 weeks. We confirmed that DA-9601 can modulate AD pathology and restore levels of *Akkermansia muciniphila* in a mouse model of AD.

## Results

### Decreasing Aβ accumulation with DA-9601 treatment

We compared four experimental groups to analyze the effect of DA-9601 in a mouse model of AD: WT (wild type), Ctrl (transgenic AD mice without DA-9601 treatment), DA_30 mg (DA-9601 administered to Tg2576 AD mice at 30 mg/kg/day), and DA_100 mg (DA-9601 administered to Tg2576 AD mice at 100 mg/kg/day).

**Fig 1:**
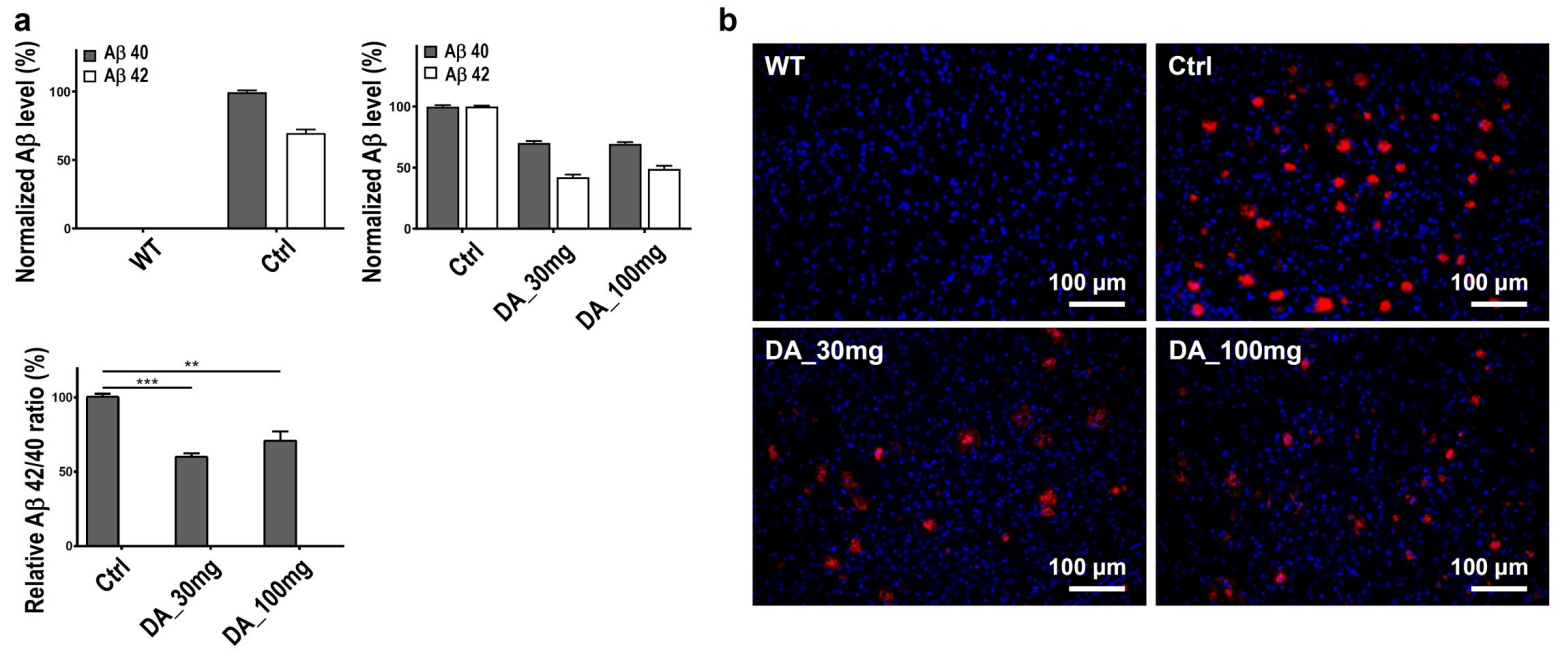
Demonstration of decreased Aβ accumulation by DA-9601 treatment. (**a**) ELISA analysis of soluble Aβ40, Aβ42, and the Aβ42/Aβ40 ratio in each experimental group. (n = 6) The first graph shows the expression levels of amyloid beta 40 and 42 in control mice, normalized to amyloid beta 40. The second graph displays the expression levels of amyloid beta 40 and 42 in experimental groups, normalized to the levels of amyloid beta 40 and amyloid beta 42 in control mice, respectively. The third graph indicates the Aβ42/Aβ40 ratio normalized to the Ctrl group. To evaluate the statistical significance, a one-way ANOVA with Tukey’s post hoc test was used (**p < 0.01, ***p < 0.001). (**b**) Representative images stained with 6E10 (red) antibody from hippocampal and cortical areas of each experimental group (n = 6).

Improvement of AD pathology through the reduction of Aβ accumulation by DA-9601 administration was confirmed by ELISA. Ctrl mice showed a higher Aβ 42/40 ratio than that of WT mice. The DA_30 mg and DA_100 mg treatments showed decreased Aβ 42/40 ratios compared with that of the Ctrl (DA_30 mg ***p<0.001; DA_100 mg **p<0.01; Fig 1A). Immunofluorescence staining also confirmed that Aβ accumulation (displayed as red dots in the Ctrl group), which was not observable in the WT group, was decreased in the DA_30 mg and DA_100 mg groups (Fig 1B).

### Reduction in microglial activation

**Fig 2:**
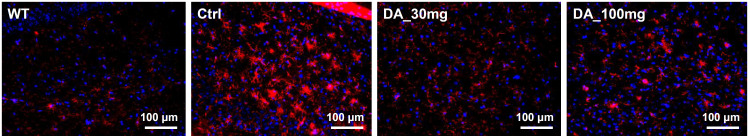
DA-9601 reduced Iba-1 expression. Representative images of immunohistochemistry for Iba-1. Compared with the Ctrl, DA_30 mg and DA_100 mg showed a decreased level of Iba-1. Data presented are representative examples from individual animals from the three experiments (6 animals per experimental condition).

AD progresses by Aβ accumulation along with microglial activation, leading to neuronal cell death. Immunostaining showed that Iba-1 expression in the Ctrl group, a marker for activated microglia, was higher than that in the WT group and significantly reduced in the DA_30 mg and DA_100 mg groups (Fig 2 and S1 Fig).

### BBB permeability

**Fig 3:**
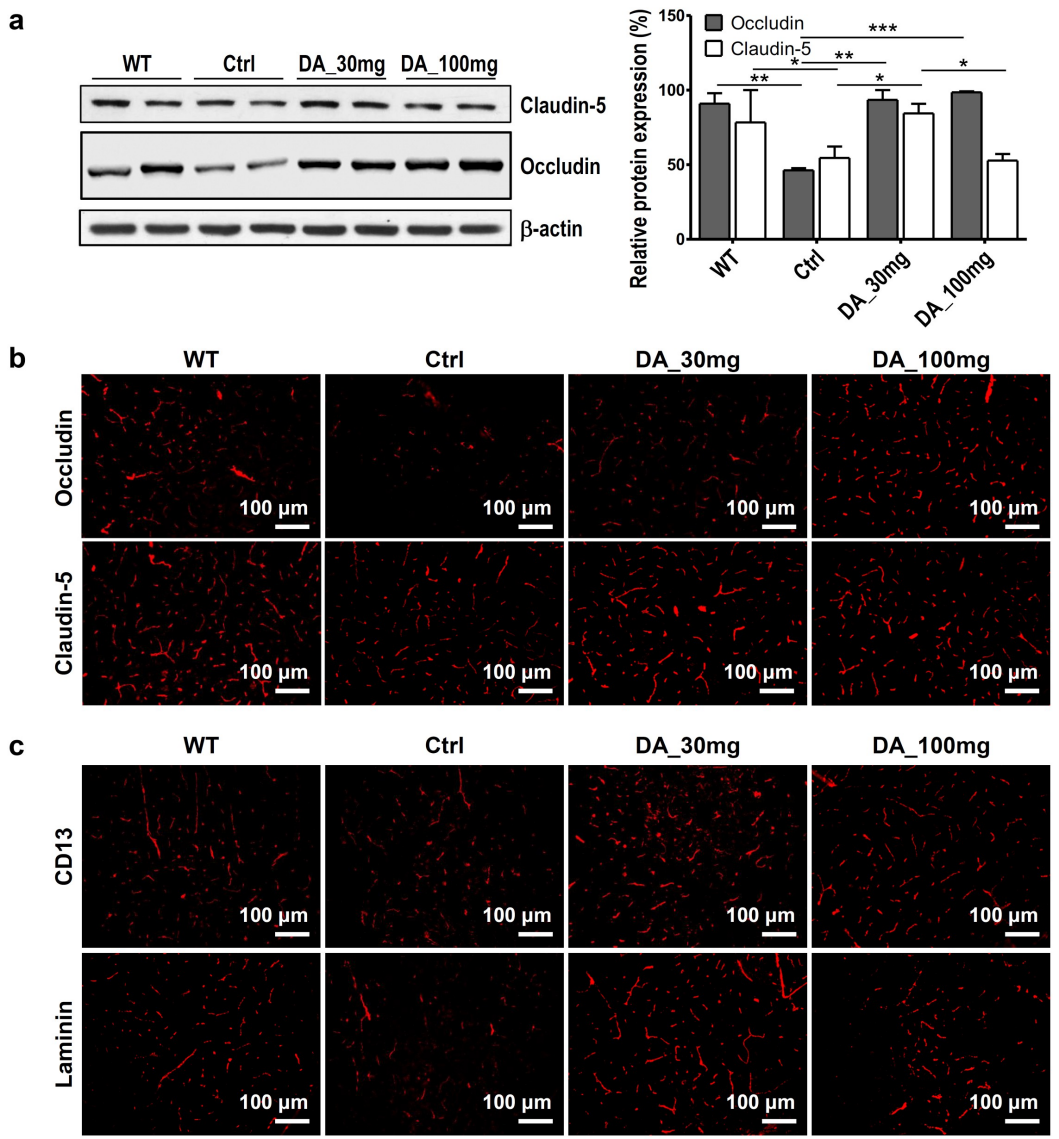
Effect of DA-9601 on the expression of endothelial occludin, claudin-5, laminin and CD13. (**a**) Immunoblots for extracted proteins from brain parenchyma. Original blots/gels are presented in S2 Fig. The levels of occludin and claudin-5 are expressed as the mean ± SEM normalized to the β-actin level in the same gel. To determine the statistically significant differences in the expression level of claudin-5 among different groups, a one-way analysis of variance and Tukey‒Kramer multiple comparisons test were used. **p < 0.01 for DA_30 mg, ***p < 0.005 for DA_100 mg. However, negligible changes in the number of endothelial claudin-5 cells with no morphological differences were observed in the DA_30 mg and DA_100 mg groups compared with the Ctrl group. (n = 6) (**b**) Representative images of occludin and claudin-5, which are tight junction proteins, from the brain parenchyma of each experimental group. (n = 6) (**c**) Representative images of pericyte coverage of CD13 (red) in the cerebral cortex of mice from each experimental group. Laminin (red) was used as an endothelial cell marker. Increased levels of Laminin and CD13 were observed in the DA_30 mg group compared with the Ctrl group. An increased number of CD13 cells was observed in the DA_100 mg group compared with the Ctrl group. Data presented are representative examples from individual animals from the three experiments (6 animals per experimental condition).

Claudin-5 is a tight junction protein in the BBB and is associated with neurodegenerative disorders such as AD. Immunoblot analysis showed that the Ctrl group expressed lower levels of claudin-5 than the WT group (*p<0.05), and claudin-5 expression in the DA_30 mg group was higher than that in the Ctrl group (*p < 0.05; Fig 3A). Full-length gels and blots are included in a Supporting Information (S2 Fig). Occludin, a protein for conjugation, showed lower expression in the Ctrl than the WT (**p<0.01). The expression of occludin was increased by administration of DA-9601 (**p < 0.01 for DA_30 mg; ***p<0.001 for DA_100 mg; Fig 3A). Consistent with the immunoblot results, IHC analysis also demonstrated that the Ctrl group expressed reduced levels of claudin-5 and occludin compared to the WT group, which were restored by DA-9601 administration (Fig 3B and S3 Fig).

The expression of laminin, which regulates the vascular integrity of the BBB, showed little difference between the Ctrl and WT groups in the IHC analysis. However, an increase in laminin expression was observed in the DA_30 mg group (Fig 3C and S3 Fig). CD13 is the protein associated with blood vessel formation, and the Ctrl group had lower expression of CD13 than the WT group. CD13 expression was increased in the DA_30 mg and DA_100 mg groups (Fig 3C and S3 Fig).

### Changes in the gut microbiome

**Fig 4:**
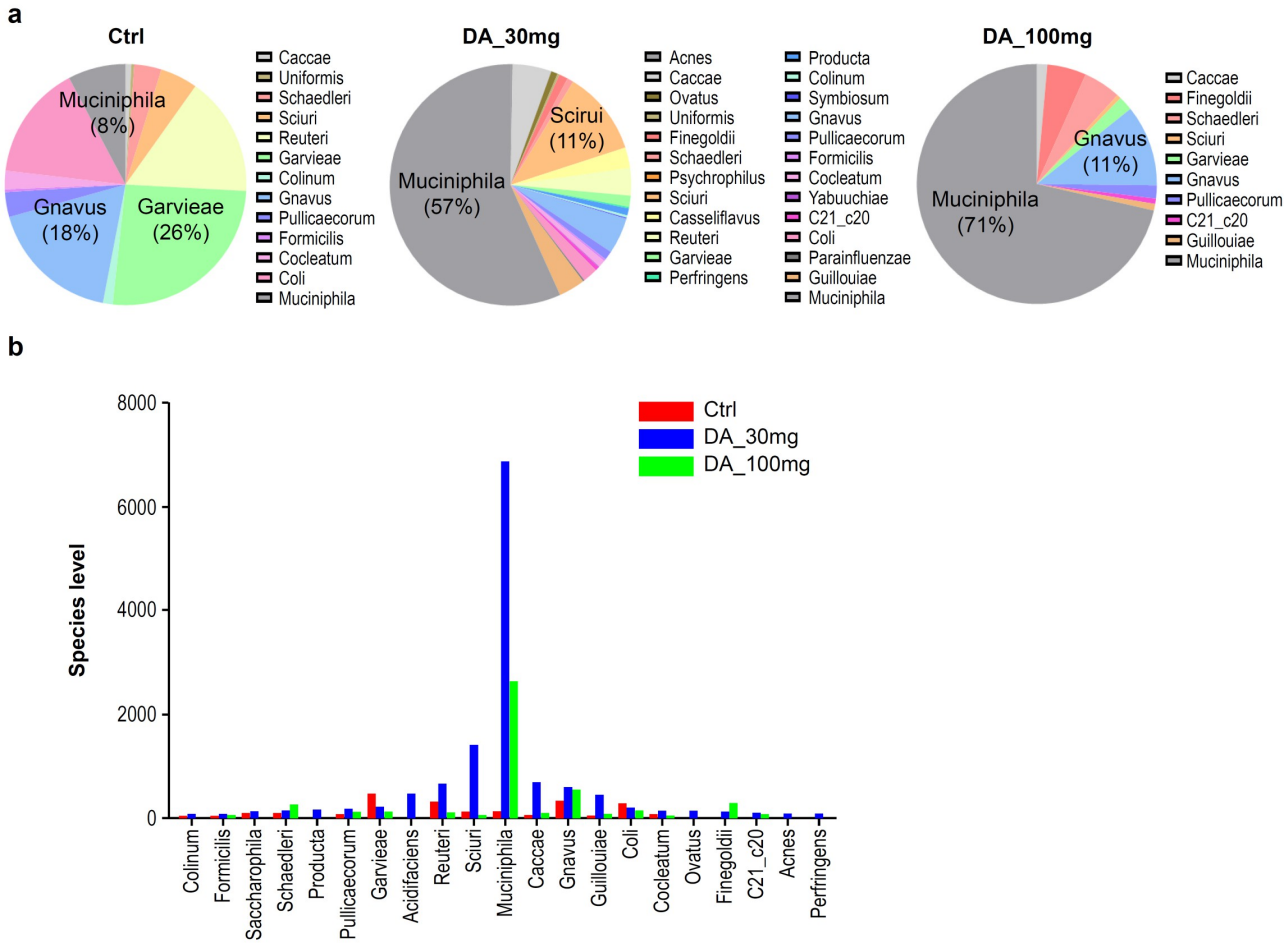
Analysis of the gut microbiome through 16S gene sequencing of fecal samples. (**a**) Relative species levels of the gut microbiome for Ctrl, DA_30 mg, and DA_100 mg. Quantities are expressed as a percentage of a specific gravity. (**b**) Species levels of the gut microbiome were compared among the Ctrl, DA_30 mg, and DA_100 mg groups. Muciniphila levels were significantly increased in the DA_30 mg and DA_100 mg groups compared with that of the Ctrl group.

Changes in the gut microbiome were analyzed using 16S gene sequencing as a microbiome profiling method (Fig 4). The Ctrl showed less gene diversity than that of the WT (S4 and S5 Figs and S1 Table). There was no difference in species diversity among DA_30 mg, DA_100 mg, and the Ctrl (Fig 4A). However, the difference in the distribution of bacterial populations between WT and the Ctrl was 0.034 (Q-value <0.05) (S4–S6 Figs and S1 Table). In particular, *Akkermansia muciniphila* increased from 10% in the Ctrl to 57% in DA_30 mg and 71% in DA_100 mg. The LDA (linear discriminant analysis) score also confirmed that *Akkermansia muciniphila* colonies were increased in the DA_30 mg and DA_100 mg groups compared with that of the Ctrl group. *Lactobacillus* and *Adlercreutzia* colonies were decreased in DA_30 mg. *Lactobacillus*, *Oscillospira*, *Streptococcus*, and *Lactococcus c*olonies were decreased in DA_100 mg (Fig 4B and S2 and S3 Tables).

### Adverse events and cognitive function

During the two-week experimental period, there were no deaths or other adverse events caused by DA-9601 administration. The increase in the body weight curve in the control and administration groups was within the normal range (S7 Fig). The novel object recognition test was used to evaluate cognitive function (S8 Fig). Ctrl mice were less likely to recognize novel objects than WT mice (***p<0.001). The DA-9601-administered groups showed a significant increase in exploratory behavior compared with that of the Ctrl group (DA_30 mg **p<0.01; DA_100 mg **p<0.01).

## Discussion

The key pathological molecule in AD is known to be Aβ, which induces neurotoxicity through self-aggregation [2–5]. Aβ is closely related to the gut microbiome through the gut-brain axis [9, 10, 23–25]. Disruption of the gut microbiome can either contribute to the onset of AD or result from its progression [41–43]; the gut microbiome of AD patients differs significantly from that of healthy individuals [44, 45]. Therefore, it can be inferred that the composition of the gut microbiome plays an important role in the accumulation of Aβ [41, 46, 47]. In particular, a reduction in *Akkermansia muciniphila* has been reported to be associated with obesity, type 2 diabetes, dysfunction of the intestinal barrier and other metabolic syndromes, all of which are key risk factors for AD [26, 28, 48, 49]. However, a few studies have also reported that *Akkermansia muciniphila* improves glucose tolerance, intestinal barrier dysfunction and dyslipidemia [27, 41].

In this work, we suggested that DA-9601 affects the gut microbiome which produces molecules that are transported to the brain. The BBB is a major selective barrier for the delivery of molecules and is affected as AD progresses. Therefore, BBB integrity was analyzed to evaluate the effect of DA-9601 on AD. We confirmed that DA-9601 improves AD pathology by reducing Aβ accumulation and microglial activation with modulation of BBB integrity. In particular, we demonstrated that *Akkermansia muciniphila* is one of the major microbiome constituents affected by AD and significantly restored by DA-9601, thus identifying it as a key element of the gut-brain axis in the context of DA-9601.

BBB permeability and microglial activation are well-known pathologic phenomena in AD [50]. Therefore, the interplay among changes in the gut microbiome, BBB permeability, and microglial activation would support the modulation of AD through the gut-brain axis [51–53]. The gut microbiome can produce Aβ-like metabolites that mimic human central nervous system Aβ, which lead to protein misfolding and microglial activation [11, 23–25]. Consequently, controlling Aβ-like proteins produced by the gut microbiome may be a promising strategy to modulate AD [44]. BBB permeability [49] and the integrity of blood vessels were evaluated by the expression of claudin-5, laminin, occludin, and CD13 [54]. Their different expression between the Ctrl and WT and recovered expression by DA-9601 indicated that DA-9601 decreased microglial activation and altered BBB permeability.

16S gene sequencing analysis was performed to identify changes in the gut microbiome that are responsible for the modulation of AD pathology by DA-9601 treatment. It was confirmed that the level of *Akkermansia muciniphila* was significantly lower in the Ctrl group and was recovered by DA-9601 treatment over 14 days. Additionally, patients with various conditions, including obesity [55–60], type 2 diabetes [61–63], atherosclerosis [64–67], and autism-related gastrointestinal disturbances [68], have also been reported to exhibit lower levels of *Akkermansia muciniphila* [69]. *Akkermansia muciniphila* can affect the brain by secreting acetylcholine into the bloodstream, which is a major neurotransmitter that controls memory and cognition [70]. Furthermore, *Akkermansia muciniphila* was reported to play a protective role by improving glucose tolerance, intestinal barrier dysfunction, and dyslipidemia in AD model mice [71].

In summary, DA-9601 improved the gut microbiome environment, particularly the abundance of *Akkermansia muciniphila*, in the mouse model of AD. *Akkermansia muciniphila* restored BBB integrity and reduced microglial activation, resulting in recovery of memory and cognitive function by reducing Aβ accumulation. Thus, targeting *Akkermansia muciniphila* could be a promising approach for the amelioration of AD [42, 43].

## Conclusions

Here, we demonstrated the interaction between changes in the gut microbiome in response to DA-9601 and AD pathology through BBB integrity and microglial activation in a mouse model of AD. DA-9601 restored the level of *Akkermansia muciniphila* and BBB integrity reduced by AD and reduced microglial activation and Aβ accumulation. Taken together, DA-9601 administration induced *Akkermansia muciniphila* restoration, which can play a critical role in AD modulation through the gut-brain axis in Tg2576 AD mice.

## Materials and methods

### Animal experiments and experimental groups

All processes for animal experiments were approved by the Institutional Animal Care and Use Committee of Seoul National University Hospital (IACUC, Approval number: 19-0126-S1A1), which is accredited by the Association for the Assessment and Accreditation of Laboratory Animal Care International. All experiments were performed in accordance with relevant guidelines and regulations. This study is reported in accordance with ARRIVE guidelines. Heterozygous Tg2576 transgenic mice were used as animal model for AD, which express human APP (from Professor Inhee Mook-Jung, Seoul National University College of Medicine). Solid feed and filtered pure water were freely offered to animals. The mice were kept in groups with a 12-hour light and dark cycle each day, and they had unrestricted access to food and water. Nine-month-old male Tg2576 mice were allocated randomly into four distinct groups: WT (n = 6), Ctrl (n = 6), DA_30 mg (n = 6), and DA_100 mg (n = 6). The humane endpoint was set when the experimental animal suffered severe pain, lost appetite for 3 days, or consumed only 40% of its diet for 7 days. However, tissue extraction was required to check molecular biological effect indicators after two weeks of DA-9601 administration to mice, which usually have a lifespan of one year, and no toxicity of DA-9601 was observed, so the animals did not reach the humane endpoint. All people participating in animal experiment completed the Preclinical Experiment Department user training and laboratory animal technique workshops (SNU 12-55-42).

### Administration of DA-9601

Dong-A Co., Ltd. (Seoul, South Korea) provided DA-9601. The dosage was set to not exceed 0.02 ml/g. Body weights were recorded just before administration for two weeks at specific time points. DA-9601 was mixed with sterile distilled water. DA-9601 was administered orally using a sonde every day for two weeks. The reference material used was sterile distilled water.

### Observing behavior and assessing lethality

The onset of toxicity and changes in general condition were monitored for 6 hours after oral administration. Abnormal behaviors were observed that could be induced by DA-9601. In the case of abnormalities, the type and severity of symptoms were recorded individually. All mice were monitored every day until they reached critical condition or death.

### Novel object recognition test

The novel object recognition test evaluates hippocampal-dependent memory performance. An open acrylic chamber (50 × 50 × 50 cm) served as the testing environment. Mice were given a one-week acclimation period, during which they were transferred from the home cage to the testing chamber once daily for 30 minutes during the first three days. Starting on the fourth day, objects were introduced into the testing chamber in the 30 minutes of acclimation process. Testing begins after a 1-week acclimatization period. Initially, mice were introduced into the chamber for a 30-minute acclimation period. Subsequently, two distinct objects were placed within the chamber; one was the object used during the acclimatization phase, while the other was a novel object. The mice were permitted to explore these objects during three separate 5-minute trials. Between each trial, the mice were returned to their home cages for a one-hour interval. After completing three trials, the mice returned to the home cage. Twenty-four hours later, they were transferred from the home cage to the testing chamber for a 30-minute acclimation period, followed by daily testing with the same two distinct objects used the previous day.

The chamber was thoroughly cleaned with ethanol, and the objects were disinfected prior to each trial to remove any odor cues. The objects were securely affixed to the bottom of the chamber to prevent displacement by the mice. The duration of time spent by the mice in the central area of the chamber (encompassing 70% of the total area) and their movement speed were measured using TOPSCSn, with all behavioral activities recorded. An object was considered to be under investigation when the mouse’s nose was within 1 cm of the object.

### Fecal sample collection from mice

Fresh fecal samples were collected from mice into sterilized EP tubes and immediately frozen with dry ice. The samples were subsequently transferred to a −80°C freezer for storage until DNA extraction.

### DNA extraction and 16S rRNA gene amplification sequence

The processes were conducted based on the author’s previous research [72]. The samples were centrifuged at 15,000 rpm and 4°C for 30 minutes to isolate the cellular pellet from the cell-free supernatant. DNA was then extracted from the pellet using the QIAamp DNA Microbiome Kit (Qiagen, Valencia, CA, USA), according to the manufacturer’s protocol.

Amplification of the 16S rRNA gene within the V3–V4 hypervariable region was conducted utilizing the primers 341F (5’ TCG TCG GCA GCG TCA GAT GTG TAT AAG AGA CAG CCT ACG GGN GGC WGC AG 3’) and 805R (5’ GTC TCG TGG GCT CGG AGA TGT GTA TAA GAG ACA GGA CTA CHV GGG TAT CTA ATC C 3’), incorporating sequences for Illumina adaptor overhangs. The resulting amplicon was purified using a magnetic bead-based system (Agencourt AMPure XP; Beckman Coulter, Brea, CA, USA). Subsequent indexing of the libraries was performed with Nextra technology, involving limited-cycle PCR, followed by an additional purification step and pooling of libraries to achieve equimolar concentrations. The final library was denatured with 0.2 N NaOH and diluted to 6 pM, incorporating a 20% PhiX control. Sequencing was executed using a 2x300 bp paired-end protocol on an Illumina MiSeq platform, in accordance with the manufacturer’s guidelines.

### Metagenome analysis

The methodologies employed were informed by the author’s prior research. Total genomic DNA was extracted from 24 fecal samples utilizing the EZNA Stool DNA kit (Omega Bio-Tek, USA), adhering strictly to the manufacturer’s protocols. DNA fragments, averaging approximately 300 bp in length, were prepared using the TruSeq DNA Sample Preparation Kit. The paired-end library was constructed with the Covaris M220 (Gene Company Limited, China). Metagenomic sequencing was performed according to the manufacturer’s guidelines on the Illumina HiSeq4000 sequencing platform (Illumina Inc., San Diego, CA, USA). Sequence reads were initially filtered, removing those with a quality score below 20 or a length under 50 bp. The remaining high-quality reads were assembled into contigs using SOAPdenovo software. Subsequently, open reading frames (ORFs) were predicted in each sample using MetaGene (http://metagene.nig.ac.jp). Annotations for the clusters of orthologous groups of proteins (COG) were determined using BLASTP (BLAST version 2.2.28+) against the eggNOG database (version 4.5), applying a significance threshold of 10^−5^.

### Bioinformatics and statistical evaluation

The processes were conducted based on the author’s previous research [72]. Initial sequence analysis, following demultiplexing, was conducted using Illumina MiSeq Reporter software. Paired-end sequences from each sample were exported in FASTA format for subsequent bioinformatics processing. Sequence analysis was performed utilizing the QIIME2 software package, and additional data analysis was carried out using Microbiome Analyst. Sequences were clustered based on a 97% similarity threshold, referencing the 2013 Green Gene ribosomal database. Sequences that did not align with this reference were assigned to new operational taxonomic units (OTUs) at 97% similarity using UCLUST. For subsequent analyses, the OTU table was subsampled to a sequencing depth of 20,000 reads per sample.

Taxonomic classification was executed following OTU aggregation at the genus level. Alpha diversity of each sample was assessed using the Shannon index. Principal coordinate analysis (PCoA) plots were generated to evaluate beta diversity, employing the Bray-Curtis dissimilarity index. Statistical significance was determined using nonparametric univariate tests, including the Mann-Whitney and Kruskal-Wallis tests, across various sampling sites and intestinal regions of the murine gut microbiome. Differences in bacterial taxa abundance were identified using linear discriminant analysis effect size (LEfSe). To correct for multiple comparisons, the false discovery rate (FDR) adjustment was applied, with significance set at a P value < 0.05.

Differentially abundant genes between mouse strains were analyzed using linear discriminant analysis (LDA) and LEfSe, with statistical significance defined by LDA values exceeding 2.5 and P values < 0.05. These methods were also employed to detect significant differences in clusters of orthologous groups (COG) and KEGG categories in the metagenomic data, with similar significance criteria applied.

All data presented in the figures are expressed as mean ± standard deviation. Experimental comparisons were conducted using the Newman-Keuls post hoc test following one-way ANOVA. Statistical analyses were performed using Prism 5 software (GraphPad Software Inc., La Jolla, CA, USA), with significance denoted by a P value < 0.05.

### Tissue preparation and fluorescent immunohistochemical analysis

The processes were conducted to minimize suffering and distress in animals based on the author’s previous research [72]. For immunohistochemical analysis, mice were anesthetized and subjected to perfusion via the heart with 10 ml of cold saline followed by 4% paraformaldehyde in 0.1 M phosphate-buffered saline (PBS). The brain was promptly excised, fixed in 4% paraformaldehyde for 48 hours, and then transferred to a 30% sucrose solution. Brains were stored at 4°C until sectioning. Frozen sections, cut to a thickness of 10 μm, were prepared. Free-floating sections were rinsed with PBS, then incubated in a blocking solution comprising 0.3% Triton X-100 and 10% normal serum in PBS for 1 hour at room temperature. Sections were subsequently incubated overnight at 4°C with primary antibodies: claudin-5 (1:50; Santa Cruz Biotechnology, Santa Cruz, CA, USA), occludin (1:50; Santa Cruz Biotechnology, Santa Cruz, CA, USA), CD13 (1:50; Abcam, Cambridge, UK), laminin (1:100; Santa Cruz Biotechnology, Santa Cruz, CA, USA), and β-amyloid (1:50; Santa Cruz Biotechnology, Santa Cruz, CA, USA). Following primary antibody incubation, sections were washed with PBS and incubated with Cy3-conjugated anti-mouse IgG and Cy3-conjugated anti-rabbit IgG (1:100; Jackson ImmunoResearch Laboratories) for 2 hours. The stained sections, with occludin, claudin-5, CD13, and laminin (red) or DAPI (blue), were examined using an upright microscope (Ni-E, Nikon Corporation, Tokyo, Japan).

### Quantitative assessment of Aβ accumulation

Sandwich-type Aβ enzyme-linked immunosorbent assays (ELISA) were conducted to quantify the levels of Aβ 1–40 and Aβ 1–42 in whole brain extracts from six mice per experimental group. Supernatant fractions were analyzed using ELISA kits specific for Aβ 1–40 and Aβ 1–42, following the manufacturer’s protocols (KMB 3481 and KMB 3441, respectively; Invitrogen, Camarillo, CA, USA) to assess Aβ accumulation.

Monoclonal antibodies specific to mouse Aβ 1–40 and Aβ 1–42 were immobilized on microplates. Fifty microliters of either standard or sample solutions were aliquoted into the center of each well, followed by the addition of 50 μl of assay buffer for dilution. The plates were incubated at room temperature for 3 hours, then subjected to five washes. Subsequently, 100 μl of Aβ 1–40 or Aβ 1–42 conjugate was added to each well, followed by a further 1-hour incubation and repeated washes. The reaction was then developed by incubating the wells with 100 μl of substrate solution for 30 minutes, and the reaction was terminated with an inhibitor solution. Each assay was conducted in duplicate to ensure reproducibility. Optical densities at 450 nm (OD450) were measured using a microplate reader (Multiskan EX; Thermo Electron Corporation, Vantaa, Finland). Quantification of Aβ 1–40 and Aβ 1–42 levels was performed by referencing a standard curve.

### Western blot analysis and protein extraction

The processes were conducted based on the author’s previous research [72]. Brains from Tg2576 mice were excised, snap-frozen in liquid nitrogen, and subsequently stored at -80°C until protein extraction. Protein extraction was performed using freshly prepared RIPA buffer (radioimmunoprecipitation assay buffer, Thermo Scientific, Waltham, MA, USA) supplemented with protease inhibitors (Roche, NJ, USA). Protein concentration was determined via a bicinchoninic acid (BCA) assay (Pierce, Rockford, IL, USA). For Western blot analysis, 40 μg of protein per sample were resolved by sodium dodecyl sulfate‒polyacrylamide gel electrophoresis (SDS‒PAGE) using 4–15% Novex NuPage Bis-Tris gels (Invitrogen, Mount Waverley, Australia) and transferred to polyvinylidene fluoride (PVDF) membranes (Millipore, Bedford, MA, USA). Membranes were blocked with 5% nonfat milk powder in 1× TBST (Tris-buffered saline with 0.1% v/v Tween-20) for 1 hour at room temperature. The blots were then incubated overnight at 4°C with diluted primary antibodies, including occludin (1:200; Santa Cruz Biotechnology, Santa Cruz, CA, USA), claudin-5 (1:200; Santa Cruz Biotechnology, Santa Cruz, CA, USA), Iba1 (1:200; Santa Cruz Biotechnology, Santa Cruz, CA, USA), and anti-β-actin (1:200; Santa Cruz Biotechnology, Santa Cruz, CA, USA). Secondary antibody incubation was performed with anti-rabbit antibody (1:3000; GE Healthcare, NJ, USA) or horseradish peroxidase-conjugated anti-mouse antibody. Blots were developed using enhanced chemiluminescence (ECL) solution (Advansta, CA, USA). Band intensities were quantified using ImageJ software, with results normalized to β-actin. Data presented represent three independent experiments.

## Supporting information

S1 Table Changes in genus diversity by DA-9601 treatment in the mice model of AD. WT (Group1), Ctrl (Group2), DA_30mg (Group3), and DA_100mg (Group4) were compared. Ctrl showed reduced gene diversity compared to WT (*p<0.05), and it can be seen that DA-9601 treatment did not affect the total gene diversity.
(DOCX)

S2 Table Species levels of the gut microbiome were compared among the Ctrl, DA_30 mg, and DA_100 mg groups. Muciniphila levels were significantly increased in the DA_30 mg and DA_100 mg groups compared with that of the Ctrl group.
(DOCX)

S3 Table Raw data for demonstration of decreased Aβ accumulation by DA-9601 treatment. Table indicates normalized Aβ40, Aβ42, and the Aβ42/Aβ40 ratio in each experimental group in Fig 1A.
(DOCX)

S1 Fig Quantitative analysis for IHC. WT, Ctrl, DA_30mg, and DA_100mg experimental groups were compared to evaluate microglial activation. The level of Iba-1 expression is normalized to WT.
(DOCX)

S2 Fig Full-length of western blots. Full-length immunoblots for extracted proteins from brain parenchyma. Gel/membrane edges are not visible because of the limitation from the equipment.
(DOCX)

S3 Fig Quantitative analysis for IHC. WT, Ctrl, DA_30mg, and DA_100mg experimental groups were compared to evaluate BBB permeability. The level of expression is normalized to WT.
(DOCX)

S4 Fig Full data of microbiome profiling for species level. WT, Ctrl, DA_30mg, and DA_100mg were compared. *Muciniphila* levels in DA_30mg and DA_100mg are significantly increased compared to that of Ctrl.
(DOCX)

S5 Fig Comparison for microbiome profiling in species level. Ctrl, DA_30mg, and DA_100mg were compared. *Muciniphila* levels in DA_30mg and DA_100mg are significantly increased compared to that of Ctrl.
(DOCX)

S6 Fig Gene distribution chart by DA-9601 treatment in the mice model of AD. WT, Ctrl, DA_30mg, and DA_100mg were compared. *Muciniphila* levels in DA_30mg and DA_100mg are significantly increased compared to that of Ctrl.
(DOCX)

S7 Fig Changes in body weight by DA-9601 treatment in the mice model of AD. Dosage is set to control, 30 mg/kg/day, and 100 mg/kg/day for male, which are set by per body weight (kg) respectively. For every animal, body weights were measured just before the administration, twice a week at certain time during 2 weeks. In all figures, brain tissues of normal mice were used as control. n = 6 samples per group.
(DOCX)

S8 Fig Effect of DA-9601 on novel object recognition in the mouse model of AD. Quantification of the time for exploring the object zone. During the familiarization phase, the number of entries and the time spent in the area were not affected by DA-9601 administration. However, during the testing phase, administration of DA-9601 significantly increased the number of entries, and the time spent in the familiar object zone. Ctrl mice showed decreased exploration time for familiar objects compared with WT mice. The results are expressed as the mean ± SD, n = 6, *P<0.05, **P<0.01, ***P<0.001 compared with Ctrl; **P<0.01 compared with DA_30 mg; and **P<0.01 compared with DA_100 mg.
(DOCX)

S1 Raw images (PDF)

## Abbreviations

AD: (Alzheimer’s disease)
BBB: (blood‒brain barrier)
Aβ: (amyloid beta)
ELISA: (enzyme-linked immunoassay)
IHC: (immunohistochemistry)
DA-9601: (*Artemisia asiatica ex*)
