# Supplementary material for: Effects of Artemisia asiatica ex on Akkermansia muciniphila dominance for modulation of Alzheimer’s disease in mice
Source: PLoS One. 2024 Oct 28;19(10):e0312670. doi: 10.1371/journal.pone.0312670 (PMC11516174; doi:10.1371/journal.pone.0312670)
Supplement: S1 Table — WT (Group1), Ctrl (Group2), DA_30mg (Group3), and DA_100mg (Group4) were compared. Ctrl showed reduced gene diversity compared to WT (*p<0.05), and it can be seen that DA-9601 treatment did not affect the total gene diversity. (DOCX) [file pone.0312670.s001.docx]

|  | **P-value < 0.05** | **Group1 vs Group2** | **Group1  vs  Group3** | **Group1  vs  Group4** | **Group2 vs Group3** | **Group2 vs Group4** | **Group3 vs Group4** |
| --- | --- | --- | --- | --- | --- | --- | --- |
|  | **Metrics** |  |  |  |  |  |  |
| **Alpha Diversity Rarefaction** | Observed_OTUs | 0.289 | 0.289 | 0.289 | 0.245 | 0.469 | 0.248 |
|  | Shannon | 0.480 | 0.724 | 0.724 | 0.386 | 0.773 | 0.248 |
|  | Faith_PD | 0.034 | 0.157 | 0.0771 | 0.248 | 0.773 | 0.773 |
| **Beta Diversity Distance** | Jaccard | 0.386 | 0.203 | 0.661 | 0.078 | 0.599 | 0.152 |
|  | Bray-Curtis | 0.213 | 0.363 | 0.494 | 0.545 | 0.377 | 0.824 |
|  | Unweighted_unifrac | 0.567 | 0.395 | 0.578 | 0.472 | 0.811 | 0.690 |
|  | Weighted_unifrac | 0.239 | 0.635 | 0.774 | 0.896 | 0.537 | 0.704 |
|  |  |  |  |  |  |  |  |
|  | **Q-value < 0.05** | **Group1 vs Group2** | **Group1  vs  Group3** | **Group1  vs  Group4** | **Group2 vs Group3** | **Group2 vs Group4** | **Group3 vs Group4** |
|  | **Metrics** |  |  |  |  |  |  |
| **Alpha Diversity Rarefaction** | Observed_OTUs | 0.289 | 0.289 | 0.289 | 0.245 | 0.469 | 0.248 |
|  | Shannon | 0.480 | 0.724 | 0.724 | 0.386 | 0.773 | 0.248 |
|  | Faith_PD | 0.034 | 0.157 | 0.077 | 0.248 | 0.773 | 0.773 |
| **Beta Diversity Distance** | Jaccard | 0.386 | 0.203 | 0.661 | 0.078 | 0.599 | 0.152 |
|  | Bray-Curtis | 0.213 | 0.363 | 0.494 | 0.545 | 0.377 | 0.824 |
|  | Unweighted_unifrac | 0.567 | 0.395 | 0.578 | 0.472 | 0.811 | 0.690 |
|  | Weighted_unifrac | 0.239 | 0.635 | 0.774 | 0.896 | 0.537 | 0.704 |

**S1 Table. Changes in genus diversity by DA-9601 treatment in the mice model of AD.** WT (Group1), Ctrl (Group2), DA_30mg (Group3), and DA_100mg (Group4) were compared. Ctrl showed reduced gene diversity compared to WT (*p<0.05), and it can be seen that DA-9601 treatment did not affect the total gene diversity.
