# Supplementary material for: Effects of Artemisia asiatica ex on Akkermansia muciniphila dominance for modulation of Alzheimer’s disease in mice
Source: PLoS One. 2024 Oct 28;19(10):e0312670. doi: 10.1371/journal.pone.0312670 (PMC11516174; doi:10.1371/journal.pone.0312670)
Supplement: S2 Table — Muciniphila levels were significantly increased in the DA_30 mg and DA_100 mg groups compared with that of the Ctrl group. (DOCX) [file pone.0312670.s002.docx]

|  | **Ctrl** | **DA_30mg** | **DA_100mg** |
| --- | --- | --- | --- |
| Colinum | 20 | 32 | 0 |
| Formicilis | 8 | 44 | 4 |
| Saccharophila | 65 | 82 | 0 |
| Schaedleri | 54 | 104 | 234 |
| Producta | 0 | 114 | 0 |
| Pullicaecorum | 46 | 133 | 85 |
| Garvieae | 450 | 176 | 93 |
| Acidifaciens | 0 | 441 | 0 |
| Reuteri | 281 | 629 | 68 |
| Sciuri | 91 | 1376 | 26 |
| Muciniphila | 76 | 6860 | 2606 |
| Caccae | 16 | 652 | 63 |
| Gnavus | 290 | 566 | 508 |
| Guillouiae | 11 | 407 | 40 |
| Coli | 257 | 159 | 114 |
| Cocleatum | 42 | 117 | 19 |
| Ovatus | 0 | 106 | 0 |
| Finegoldii | 0 | 96 | 256 |
| C21_c20 | 0 | 66 | 36 |
| Acnes | 0 | 36 | 0 |
| Perfringens | 0 | 30 | 0 |

**S2 Table. Species levels of the gut microbiome were compared among the Ctrl, DA_30 mg, and DA_100 mg groups.** Muciniphila levels were significantly increased in the DA_30 mg and DA_100 mg groups compared with that of the Ctrl group.
