# Supplementary material for: Effects of Artemisia asiatica ex on Akkermansia muciniphila dominance for modulation of Alzheimer’s disease in mice
Source: PLoS One. 2024 Oct 28;19(10):e0312670. doi: 10.1371/journal.pone.0312670 (PMC11516174; doi:10.1371/journal.pone.0312670)
Supplement: S3 Table — Table indicates normalized Aβ40, Aβ42, and the Aβ42/Aβ40 ratio in each experimental group in Fig 1A. (DOCX) [file pone.0312670.s003.docx]

|  | Aβ40 | | | | | | Aβ42 | | | | | |
| --- | --- | --- | --- | --- | --- | --- | --- | --- | --- | --- | --- | --- |
| WT | 0 | 0 | 0 | 0 | 0 | 0 | 0 | 0 | 0 | 0 | 0 | 0 |
| Ctrl | 96 | 100 | 104 | 96 | 99 | 102 | 64 | 67 | 79 | 64 | 68 | 76 |
|  |  |  |  |  |  |  |  |  |  |  |  |  |
|  | Aβ40 | | | | | | Aβ42 | | | | | |
| Ctrl | 97 | 100 | 104 | 99 | 103 | 96 | 102 | 98 | 101 | 99 | 98 | 102 |
| DA_30mg | 75 | 68 | 74 | 66 | 66 | 72 | 50 | 38 | 47 | 39 | 37 | 72 |
| DA_100mg | 72 | 72 | 66 | 65 | 73 | 69 | 41 | 43 | 51 | 56 | 55 | 48 |
|  |  |  |  |  |  |  |  |  |  |  |  |  |
|  | Aβ42/Aβ40 | | | | | |  | | | | | |
| Ctrl | 106.3 | 98.0 | 97.1 | 96.0 | 96.0 | 103.0 |  |  |  |  |  |  |
| DA_30mg | 66.7 | 54.9 | 60.0 | 59.1 | 56.3 | 64.8 |  |  |  |  |  |  |
| DA_100mg | 56.9 | 66.7 | 73.9 | 86.1 | 57.9 | 83.8 |  |  |  |  |  |  |

**S3 Table. Raw data for demonstration of decreased Aβ accumulation by DA-9601 treatment.** Table indicates normalized Aβ40, Aβ42, and the Aβ42/Aβ40 ratio in each experimental group.
