# Supplementary material for: Effects of Artemisia asiatica ex on Akkermansia muciniphila dominance for modulation of Alzheimer’s disease in mice
Source: PLoS One. 2024 Oct 28;19(10):e0312670. doi: 10.1371/journal.pone.0312670 (PMC11516174; doi:10.1371/journal.pone.0312670)
Supplement: S1 Fig — WT, Ctrl, DA_30mg, and DA_100mg experimental groups were compared to evaluate microglial activation. The level of Iba-1 expression is normalized to WT. (DOCX) [file pone.0312670.s004.docx]

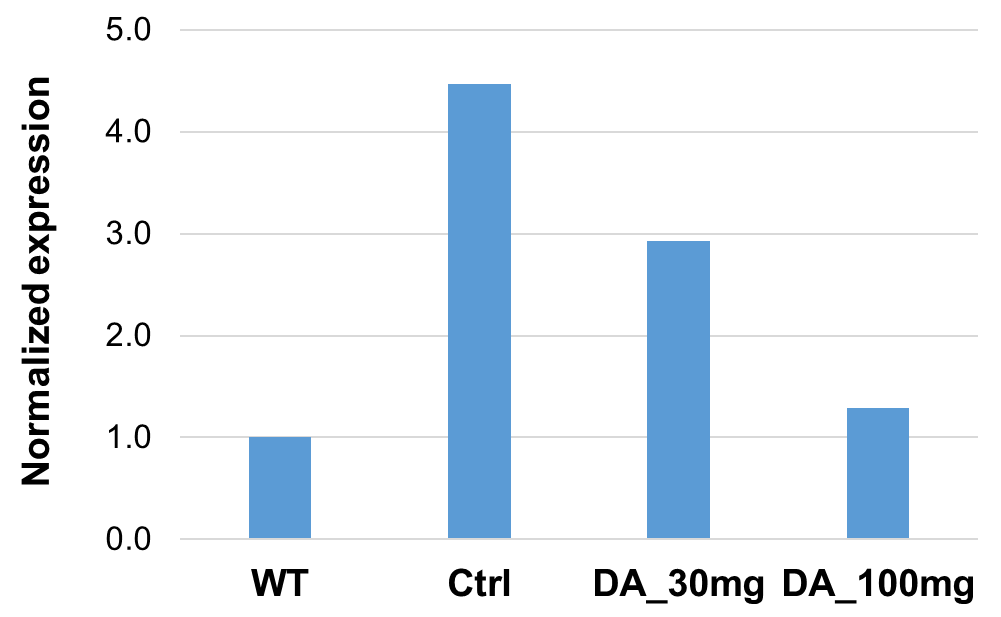


**S1 Fig. Quantitative analysis for IHC.** WT, Ctrl, DA_30mg, and DA_100mg experimental groups were compared to evaluate microglial activation. The level of Iba-1 expression is normalized to WT.
