# Supplementary material for: Effects of Artemisia asiatica ex on Akkermansia muciniphila dominance for modulation of Alzheimer’s disease in mice
Source: PLoS One. 2024 Oct 28;19(10):e0312670. doi: 10.1371/journal.pone.0312670 (PMC11516174; doi:10.1371/journal.pone.0312670)
Supplement: S2 Fig — Full-length immunoblots for extracted proteins from brain parenchyma. Gel/membrane edges are not visible because of the limitation from the equipment. (DOCX) [file pone.0312670.s005.docx]

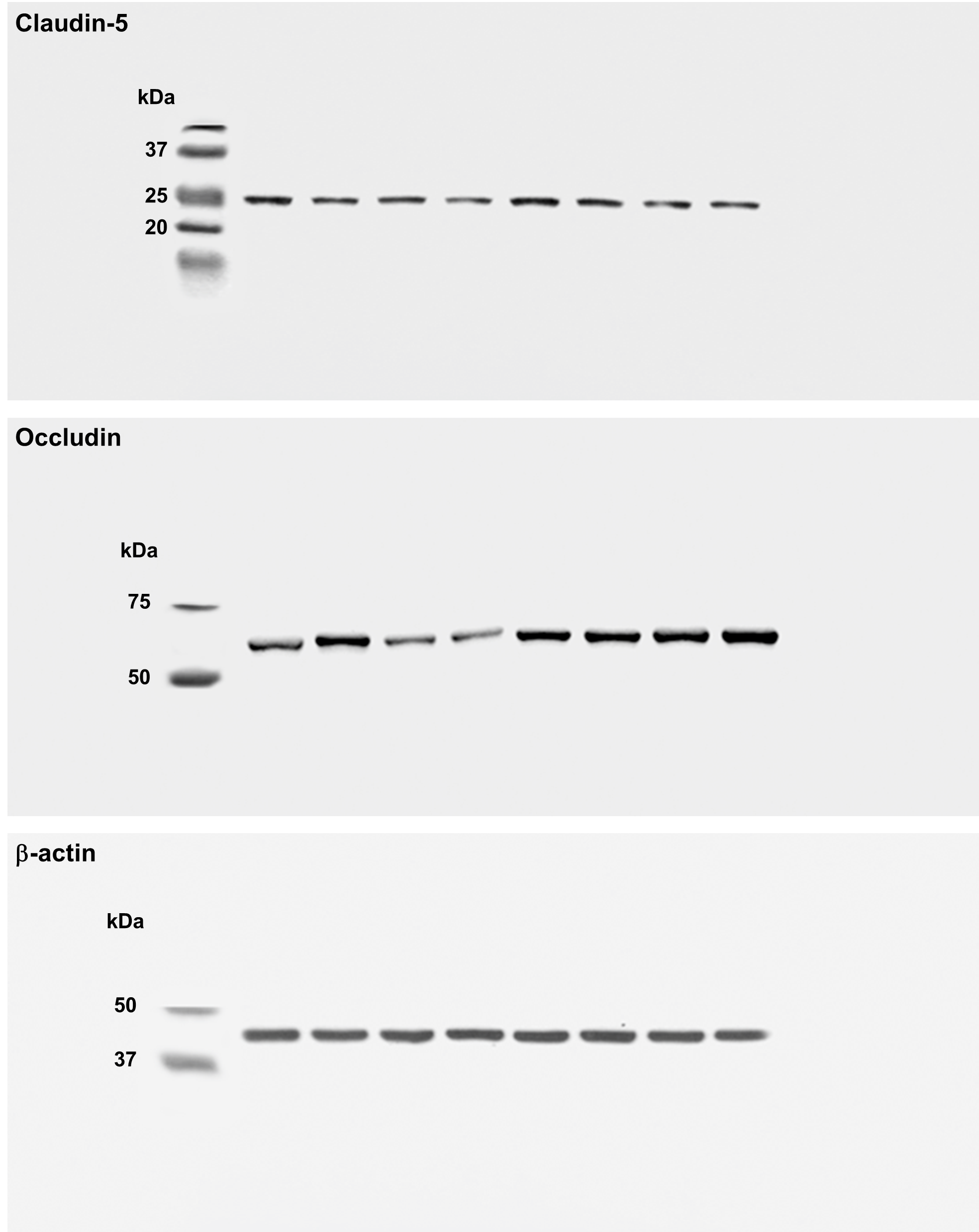


**S2 Fig. Full-length of western blots.** Full-length immunoblots for extracted proteins from brain parenchyma. Gel/membrane edges are not visible because of the limitation from the equipment.
