# Supplementary material for: Effects of Artemisia asiatica ex on Akkermansia muciniphila dominance for modulation of Alzheimer’s disease in mice
Source: PLoS One. 2024 Oct 28;19(10):e0312670. doi: 10.1371/journal.pone.0312670 (PMC11516174; doi:10.1371/journal.pone.0312670)
Supplement: S6 Fig — WT, Ctrl, DA_30mg, and DA_100mg were compared. Muciniphila levels in DA_30mg and DA_100mg are significantly increased compared to that of Ctrl. (DOCX) [file pone.0312670.s009.docx]

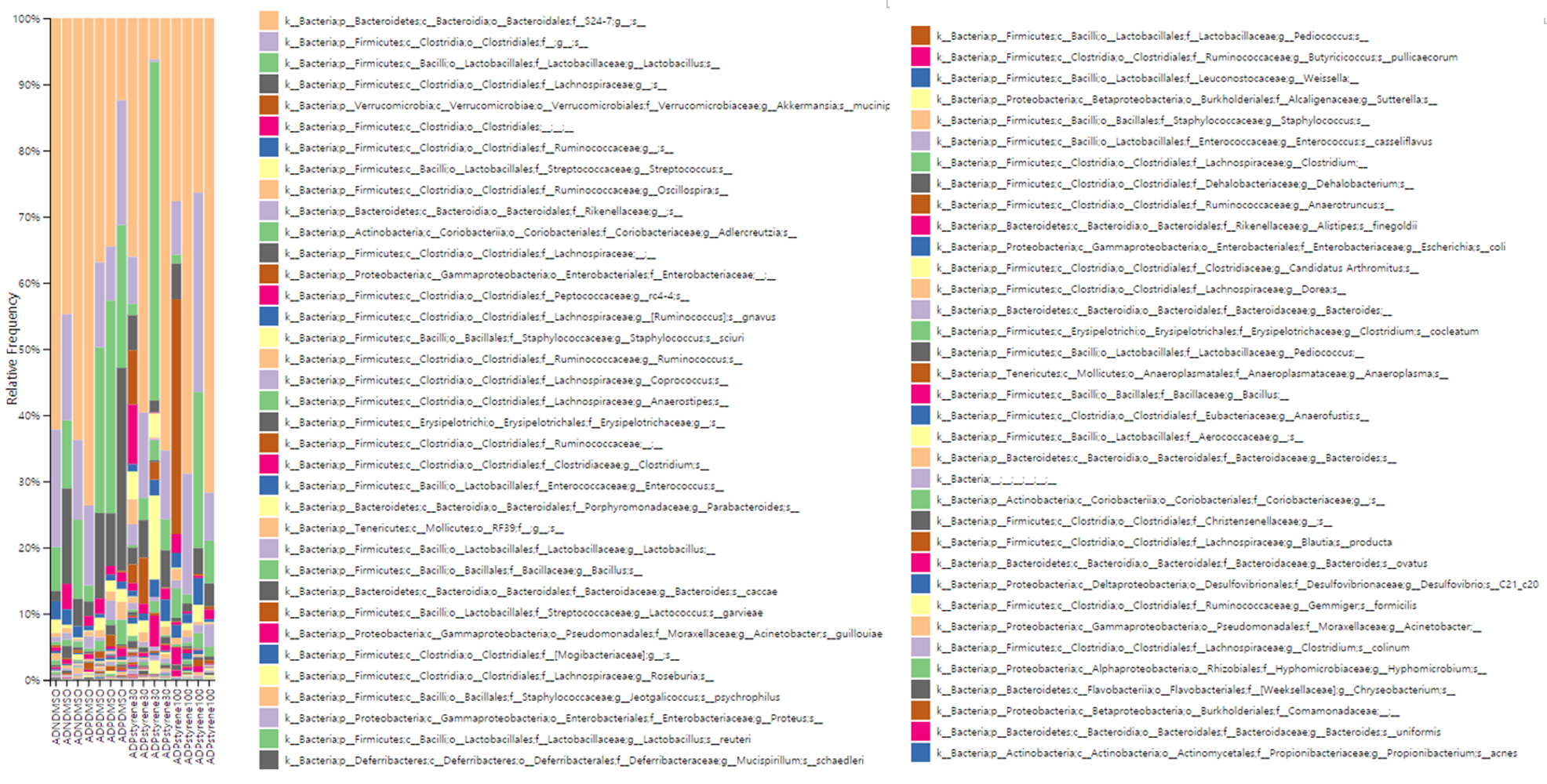


**S6 Fig. Gene distribution chart by DA-9601 treatment in the mice model of AD.** WT, Ctrl, DA_30mg, and DA_100mg were compared. *Muciniphila* levels in DA_30mg and DA_100mg are significantly increased compared to that of Ctrl.
