# Supplementary material for: Effects of Artemisia asiatica ex on Akkermansia muciniphila dominance for modulation of Alzheimer’s disease in mice
Source: PLoS One. 2024 Oct 28;19(10):e0312670. doi: 10.1371/journal.pone.0312670 (PMC11516174; doi:10.1371/journal.pone.0312670)
Supplement: S7 Fig — Dosage is set to control, 30 mg/kg/day, and 100 mg/kg/day for male, which are set by per body weight (kg) respectively. For every animal, body weights were measured just before the administration, twice a week at certain time during 2 weeks. In all figures, brain tissues of normal mice were used as control. n = 6 samples per group. (DOCX) [file pone.0312670.s010.docx]

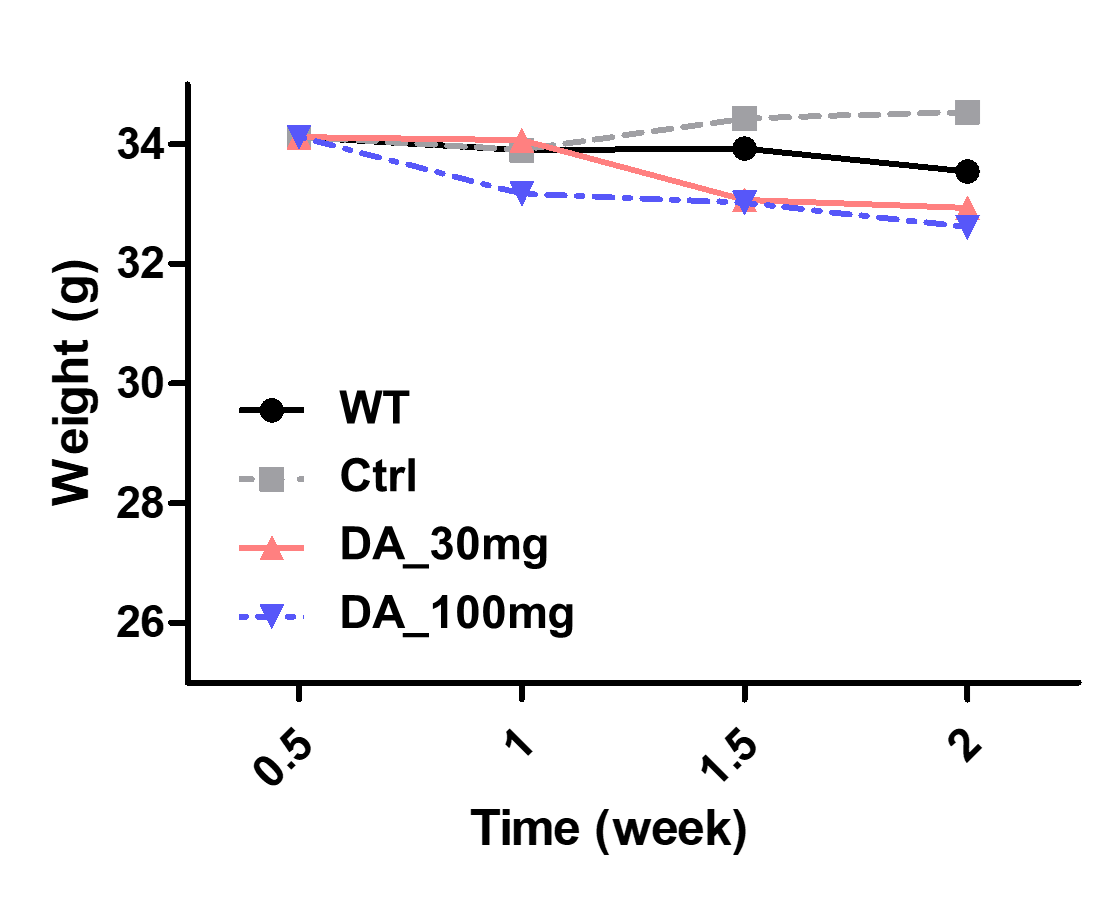


**S7 Fig. Changes in body weight by DA-9601 treatment in the mice model of AD.** Dosage is set to control, 30 mg/kg/day, and 100 mg/kg/day for male, which are set by per body weight (kg) respectively. For every animal, body weights were measured just before the administration, twice a week at certain time for 2 weeks. In all figures, brain tissues of normal mice were used as control. n = 6 samples per group.
