# Supplementary material for: Effects of Artemisia asiatica ex on Akkermansia muciniphila dominance for modulation of Alzheimer’s disease in mice
Source: PLoS One. 2024 Oct 28;19(10):e0312670. doi: 10.1371/journal.pone.0312670 (PMC11516174; doi:10.1371/journal.pone.0312670)
Supplement: S8 Fig — Quantification of the time for exploring the object zone. During the familiarization phase, the number of entries and the time spent in the area were not affected by DA-9601 administration. However, during the testing phase, administration of DA-9601 significantly increased the number of entries, and the time spent in the familiar object zone. Ctrl mice showed decreased exploration time for familiar objects compared with WT mice. The results are expressed as the mean ± SD, n = 6, *P<0.05, **P<0.01, ***P<0.001 compared with Ctrl; **P<0.01 compared with DA_30 mg; and **P<0.01 compared with DA_100 mg. (DOCX) [file pone.0312670.s011.docx]

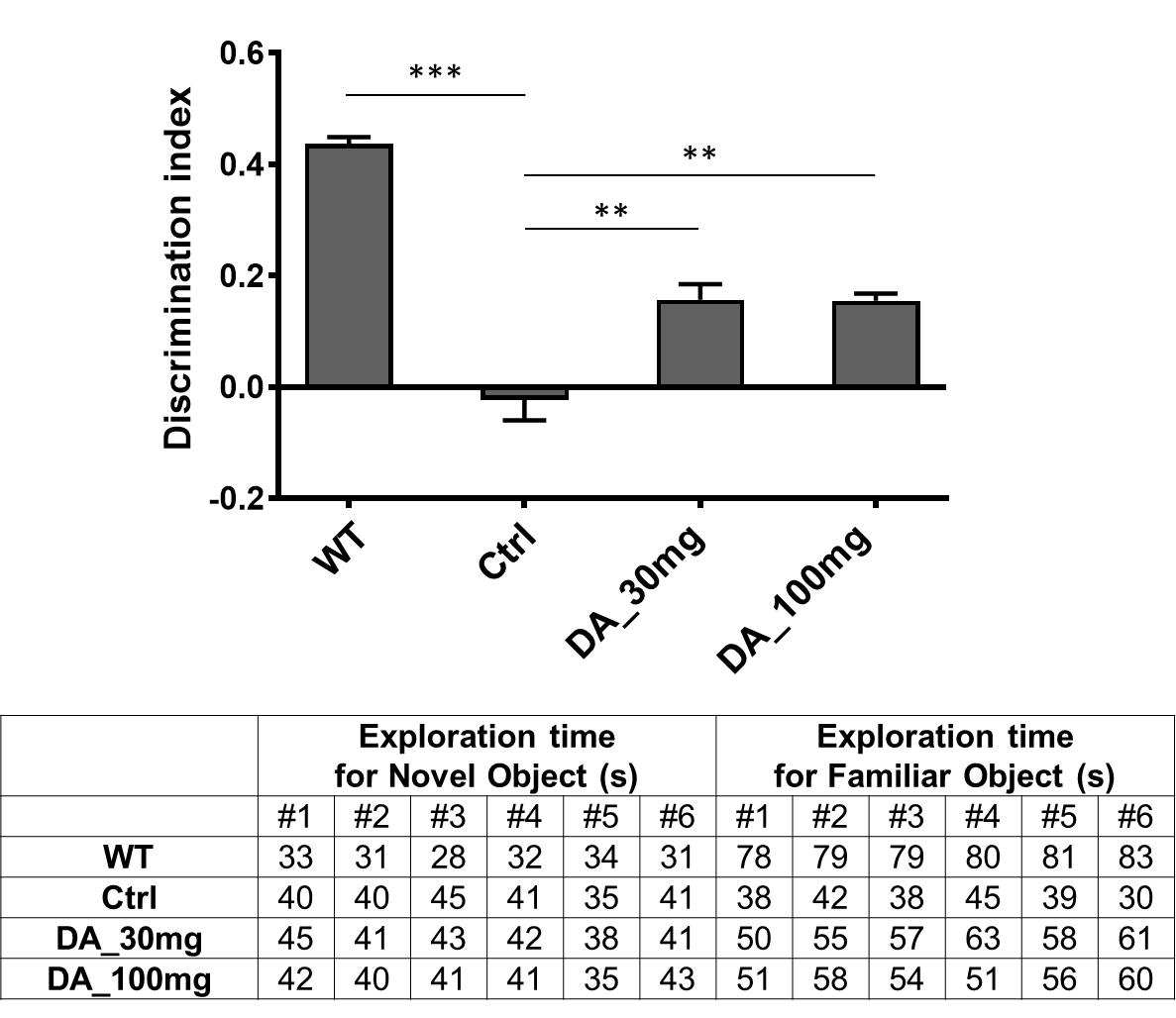


**S8 Fig. Effect of DA-9601 on novel object recognition in the mouse model of AD.** Quantification of the time for exploring the object zone. Ctrl mice showed decreased exploration time for novel objects compared with WT mice, whereas administration of DA-9601 significantly increased the time spent in the novel object zone. Discrimination index is calculated by (Novel Object Time – Familiar Object Time)/(Novel Object Time + Familiar Object Time) which is a metric that quantitatively assesses the ability to distinguish between a novel object and a familiar object. This value indicates the preference for the novel object: a score greater than 0 suggests a preference for the new object, and a negative score indicates a preference for the familiar object. It allows for the evaluation of cognitive functions. The results are expressed as the mean ± SD, n=6, *P<0.05, **P<0.01, ***P<0.001 compared with Ctrl; **P<0.01 compared with DA_30 mg; and **P<0.01 compared with DA_100 mg.
