# Supplementary figures and images for: Effects of Artemisia asiatica ex on Akkermansia muciniphila dominance for modulation of Alzheimer’s disease in mice
Source: PLoS One. 2024 Oct 28;19(10):e0312670. doi: 10.1371/journal.pone.0312670 (PMC11516174; doi:10.1371/journal.pone.0312670)

# Claudin-5

kDa

37

25

20

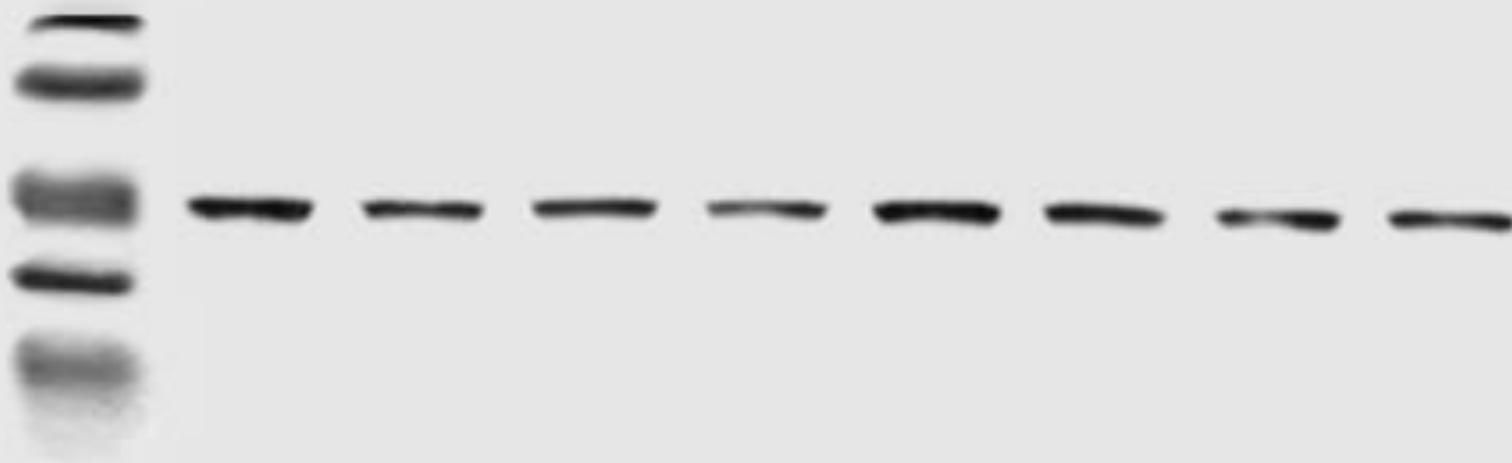

# Occludin

kDa

75

50

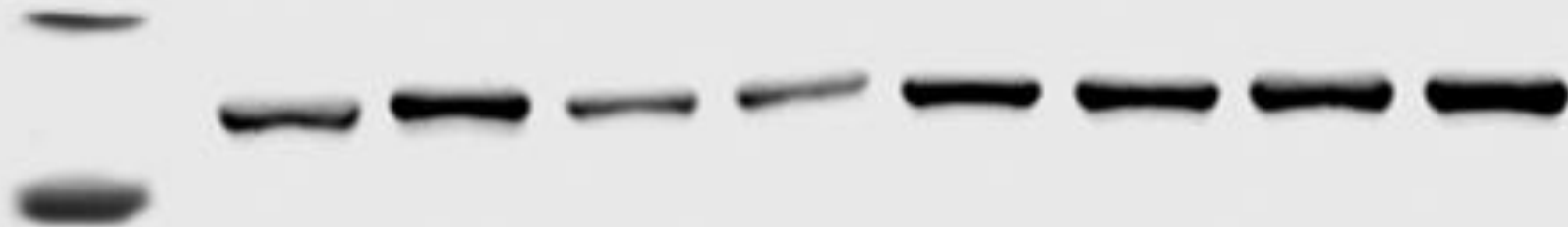

**$\beta$ -actin**

**kDa**

**50**

**37**

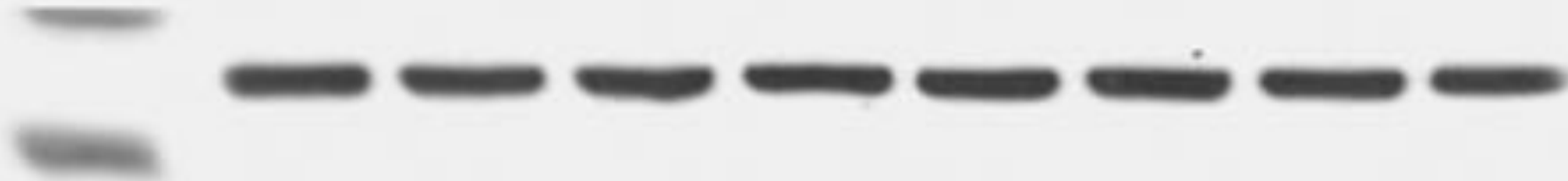

Supplement: S1 Raw images — (PDF) [file pone.0312670.s012.pdf]
